# Supplementary material for: Structural basis for excitatory neuropeptide signaling
Source: Nat Struct Mol Biol. 2024 Feb 9;31(4):717–26. doi: 10.1038/s41594-023-01198-y (PMC11026163; doi:10.1038/s41594-023-01198-y)
Supplement: Supplementary file 1 — Supplementary notes and Table 1. [file 41594_2023_1198_MOESM1_ESM.pdf]

nature structural & molecular biology

Article https://doi.org/10.1038/s41594-023-01198-y

Structural basis for excitatory neuropeptide signaling

In the format provided by the authors and unedited

Supplementary information

Structural basis for excitatory neuropeptide signaling

Valeria Kalienkova, Mowgli Dandamudi, Cristina Paulino, Timothy Lynagh

Contents:

Supplementary notes	Page 2
Table S1	Page 3

Supplementary notes

Malacoceros fuliginosus FaNaC1 coding sequence (blue font, after Genbank ON156825.1 but with silent substitutions to remove internal restriction sites) within custom oocyte expression vector (black font).

ATTTAGGTGACACTATAGAATACAAGCTTGCTTGTTCTTTTTGCAGAAGCTCAGAATAAACGCTCAACTTTGGCGTCGACACCATGGCGATTCGTGATGTAATGAC
AAAATTTGCTGAACAGACTACTATGCATGGCGTGCCAAAGGTCATTAATGCCAAATCAAGCATGGGTCGTTTGTTCTGGAGTTTAGTCTGTCTGGCAGCCGGAGCT
ATGTTTTGTCTACAAATGTCTGAAGTTTTGCAGAGATATTTCAGTTACCCAAAGAAAGTGACTGTTGAAGTCGTCCCCACTCCAGTGCCGTTTCCAAGTATCAGCA
TCTGCAACATGCGAAACCTAGATGTTCACATTCTCAACACTCTCAACCGAATGTTCATAGAAGACGACAGACCATTCAGCAATATCAATAAATCGGAACACGAATT
TATCCGAGCTTACATGAAAAAGGTGGCTAAATATGCCCCACTCTTTTGGAATTACCAAGATGAGTACCCAGAAGTCTTTCAAGAGATATTTAGTCGAACTACTTTC
AGTGCAAACATTGATCCGGAGGTAATCGCATTGGCCGCTGTTCAGCTTGAGGGCTTTGTTGTCAATTGCCATTACGCTGGACATCGGTGCAATAAGACTCGAGATT
TCTATCGGTTCTTTGATCCATACTATTTCAATTGCTTCACTTATAAAGCTCACGAACCGACAGACATCGAGGATAATTTATCAGAAGGGATAGAAAATGGGTGGTC
ATCTATTTTACTAAGTGGGAGTGGCATGTTGGATAAGAACGATGAGATTAGAATGTTGCCAGGATTACATGAGTGGCGAAGTGCTGTATCTGCCAGTGAAGGAGTA
AGGGTTGTTATACATCCACCGAGTACAACCCCGTACCCATTCACAGAGGGATATGATGTTCCACCCGGGTTTTCAGCTTCATTTGGCATTCATCCTAGACGTAATA
TCAGAATAGGACCTCCACATGGAAACTGTTCTGATAAGAATCCATTCGGTGATGGAACTGAGCGGTACAGACTGATGGCATGTCAGAAGATGTGCATGCAACATTA
TATTGTAGAAACATGCGGTTGTGCGGATGTTGGCCTCCCGAAACTACCCTTACAAGCTAATATCTCATGGTGTAGAGACGATGATAACTTTCCTGATGAGTGTATG
TTTACCGCTTCAGAGGAGTGCTTACAATTGCTAATGCAACTGCACAACAGGATTAAATGTGCTCGATCAATAAAATCTAAAATTACTAAAAACACAACTGCCATGG
AAGCATGTAATTGTTTCCCACCTTGTGATGAGGTCAGCTATGACGTGAGTTATTCACTTTCAAAATGGCCGTCAGCAGGATATGAAGGGGATGCTGCATACTTTGA
TGTATTTGGAATTGAAAAGTTCAATGAGAGGTTCAATAAAACTGGCACACAGGGCAAATATGAACTATTTACAAAGTATTTCAATGTCTCAAATCGTGAGGAGTCA
ATGAAAGATTTCGCTCGCTTGAATGTTTACATAGCTGACAGTAATGTTGTGAAAACTCAAGAATCTGAGGATTACACAAGAAATCAATTGGTGAGTGATATTGGGG
GTCAGCTAGGTCTCTGGGTCGGCATATCTCTTATCACCCTAGCCGAGGTGCTTGAACTAATTATTGATCTGTTTCGTTTATTCTCCAAACATACCTACCGAAGCGT
TCCAGTCATTCGACAAAGTATAAAATATAAAGACAAGCGGAACGGTGCAGAAATGAACTATGACACAAGATACAGTCAGTCAAATGGCGGACCTCATGCTCGTTAT
TTACATCATGGACACTCAATACCAAAGCATCCGCCAGAGTTACCTGATACAAGTTTGGATCCGAGCAGAAGCTCATCAGTGAGGAAGATCTCTAAGGTTACCACTA
AACCAGCCTCAAGAACACCCGAATGGAGTCTCTAAGCTACATAATACCAACTTACACTTTACAAAATGTTGTCCCCCAAAATGTAGCCATTCGTATCTGCTCCTAA
TAAAAAGAAAGTTTCTTCACATTCTAAAAAAAAAAAAAAAAAAAAAAAAAAAAAACGAATTCGTAATCATGTCATAGCTGTTTCCTGTGTGAAATTGTTATCCGCT
CACAATTCCACACAACATACGAGCCGGAAGCATAAAGTGTAAAGCCTGGGGTGCCTAATGAGTGAGCTAACTCACATTAATTGCGTTGCGCTCACTGCCCGCTTTC
CAGTCGGGAAACCTGTCGTGCCAGCTGCATTAATGAATCGGCCAACGCGCGGGGAGAGGCGGTTTGCGTATTGGGCGCTCTTCCGCTTCCTCGCTCACTGACTCGC
TGCGCTCGGTCGTTCGGCTGCGGCGAGCGGTATCAGCTCACTCAAAGGCGGTAATACGGTTATCCACAGAATCAGGGGATAACGCAGGAAAGAACATGTGAGCAAA
AGGCCAGCAAAAGGCCAGGAACCGTAAAAAGGCCGCGTTGCTGGCGTTTTTCCATAGGCTCCGCCCCCCTGACGAGCATCACAAAAATCGACGCTCAAGTCAGAGG
TGGCGAAACCCGACAGGACTATAAAGATACCAGGCGTTTCCCCCTGGAAGCTCCCTCGTGCGCTCTCCTGTTCCGACCCTGCCGCTTACCGGATACCTGTCCGCCT
TTCTCCCTTCGGGAAGCGTGGCGCTTTCTCATAGCTCACGCTGTAGGTATCTCAGTTCGGTGTAGGTCGTTCGCTCCAAGCTGGGCTGTGTGCACGAACCCCCCGT
TCAGCCCGACCGCTGCGCCTTATCCGGTAACTATCGTCTTGAGTCCAACCCGGTAAGACACGACTTATCGCCACTGGCAGCAGCCACTGGTAACAGGATTAGCAGA
GCGAGGTATGTAGGCGGTGCTACAGAGTTCTTGAAGTGGTGGCCTAACTACGGCTACACTAGAAGAACAGTATTTGGTATCTGCGCTCTGCTGAAGCCAGTTACCT
TCGGAAAAAGAGTTGGTAGCTCTTGATCCGGCAAACAAACCACCGCTGGTAGCGGTGGTTTTTTTGTTTGCAAGCAGCAGATTACGCGCAGAAAAAAAGGATCTCA
AGAAGATCCTTTGATCTTTTCTACGGGGTCTGACGCTCAGTGGAACGAAAACTCACGTTAAGGGATTTTGGTCATGAGATTATCAAAAAGGATCTTCACCTAGATC
CTTTTAAATTAAAAATGAAGTTTTAAATCAATCTAAAGTATATATGAGTAAACTTGGTCTGACAGTTACCAATGCTTAATCAGTGAGGCACCTATCTCAGCGATCT
GTCTATTTCGTTCATCCATAGTTGCCTGACTCCCCGTCGTGTAGATAACTACGATACGGGAGGGCTTACCATCTGGCCCCAGTGCTGCAATGATACCGCGAGACCC
ACGCTCACCGGCTCCAGATTTATCAGCAATAAACCAGCCAGCCGGAAGGGCCGAGCGCAGAAGTGGTCCTGCAACTTTATCCGCCTCCATCCAGTCTATTAATTGT
TGCCGGGAAGCTAGAGTAAGTAGTTCGCCAGTTAATAGTTTGCGCAACGTTGTTGCCATTGCTACAGGCATCGTGGTGTCACGCTCGTCGTTTGGTATGGCTTCAT
TCAGCTCCGGTTCCCAACGATCAAGGCGAGTTACATGATCCCCCATGTTGTGCAAAAAAGCGGTTAGCTCCTTCGGTCCTCCGATCGTTGTCAGAAGTAAGTTGGC
CGCAGTGTTATCACTCATGGTTATGGCAGCACTGCATAATTCTCTTACTGTCATGCCATCCGTAAGATGCTTTTCTGTGACTGGTGAGTACTCAACCAAGTCATTC
TGAGAATAGTGTATGCGGCGACCGAGTTGCTCTTGCCCGGCGTCAATACGGGATAATACCGCGCCACATAGCAGAACTTTAAAAGTGCTCATCATTGGAAAACGTT
CTTCGGGGCGAAAACTCTCAAGGATCTTACCGCTGTTGAGATCCAGTTCGATGTAACCCACTCGTGCACCCAACTGATCTTCAGCATCTTTTACTTTCACCAGCGT
TTCTGGGTGAGCAAAAACAGGAAGGCAAAATGCCGCAAAAAAGGGAATAAGGGCGACACGGAAATGTTGAATACTCATACTCTTCCTTTTTCAATATTATTGAAGC
ATTTATCAGGGTTATTGTCTCATGAGCGGATACATATTTGAATGTATTTAGAAAAATAAACAAATAGGGGTTCCGCGCACATTTCCCCGAAAAGTGCCACCTGACG
TCTAAGAAACCATTATTATCATGACATTAACCTATAAAAATAGGCGTATCACGAGGCCCTTTCGTCTCGCGCGTTTCGGTGATGACGGTGAAAACCTCTGACACAT
GCAGCTCCCGGAGACGGTCACAGCTTGTCTGTAAGCGGATGCCGGGAGCAGACAAGCCCGTCAGGGCGCGTCAGCGGGTGTTGGCGGGTGTCGGGGCTGGCTTAAC
TATGCGGCATCAGAGCAGATTGTACTGAGAGTGCACCATTCGACGCTCTCCCTTATGCGACTCCTGCATTAGGAAGCAGCCCAGTAGTAGGTTGAGGCCGTTGAGC
ACCGCCGCCGCAAGGAATGGTGCATGCAAGGAGATGGCGCCCAACAGTCCCCCGGCCACGGGGCCTGCCACCATACCCACGCCGAAACAAGCGCTCATGAGCCCGA
AGTGGCGAGCCCGATCTTCCCCATCGGTGATGTCGGCGATATAGGCGCCAGCAACCGCACCTGTGGCGCCGGTGATGCCGGCCACGATGCGTCCGGCGTAGAGGAT
CTGGCTAGCGATGACCCTGCTGATTGGTTCGCTGACCATTTCCGGGTGCGGGACGGCGTTACCAGAAACTCAGAAGGTTCGTCCAACCAAACCGACTCTGACGGCA
GTTTACGAGAGAGATGATAGGGTCTGCTTCAGTAAGCCAGATGCTACACAATTAGGCTTGTACATATTGTCGTTAGAACGCGGCTACAATTAATACATAACCTTAT
GTATCATACACATACG

Table S1. Site-directed mutagenesis primers	
Mutant primer	Primer sequence
F97C_forward	cgaatgtgcatagaagacgacagac
F97C_reverse	ttctatgcacattcggttgagagtg
D101A_forward	gaagacgccagaccattcagc
D101A_reverse	tggtctggcgtcttctatgaac
V122A_forward	catgaaaaaggcggctaaatatgccccac
V122A_reverse	catatttagccgcctttttcatgtaagctc
V122F_forward	catgaaaaagtttgctaaatatgccccac
V122F_reverse	catatttagcaaactttttcatgtaagctc
V122Q_forward	catgaaaaagcaggctaaatatgccccac
V122Q_reverse	catatttagcctgctttttcatgtaagctc
F129C_forward	cactctgttggaattaccaagatgag
F129C_reverse	ttccaacagagtggggcatatttag
F129A_forward	ccactcgcttggaattaccaagatg
F129A_reverse	attccaagcgagtggggcatatttag
F129L_forward	ccactcctttggaattaccaagatg
F129L_reverse	attccaaaggagtggggcatatttag
F129Q_forward	ccactccagtggaattaccaagatg
F129Q_reverse	attccactggagtggggcatatttag
Q133L_forward	gaattacctagatgagtacccagaag
Q133L_reverse	actcatctaggtaattccaaaagag
Q133N_forward	gaattacaacgatgagtacccagaag
Q133N_reverse	actcatcgttgtaattccaaaagag
Q133E_forward	gaattacgaagatgagtacccagaag
Q133E_reverse	actcatcttcgtaattccaaaagag
E235A_forward	gaacgatgcgattagaatgttgccag
E235A_reverse	cattctaatcgcatcgttcttatccaac
M238C_forward	gattagatgcttgccaggattacatgag
M238C_reverse	tggcaagcatctaatctcatcgttc
M238A_forward	gattagagcgttgccaggattacatg
M238A_reverse	tggcaacgctctaatctcatcgttc
M238L_forward	gattagactgttgccaggattacatg
M238L_reverse	tggcaacagtctaatctcatcgttc
M238Q_forward	gattagacagttgccaggattacatg
M238Q_reverse	tggcaactgtctaatctcatcgttc
S282C_forward	cagcttgctttggcattcatcctag
S282C_reverse	tgccaaagcaagctgaaaaccc
H297S_forward	cctccatctggaaactgttctgataag
H297S_reverse	gtttccagatggaggtcctattctg
F431A_forward	tgcatacgctgatgtatttggaattga
F431A_reverse	tacatcagcgtatgcagcatccc
F431W_forward	tgcatactgggatgtatttggaattga
F431W_reverse	tacatcccagtatgcagcatccc
F431Q_forward	tgcataccaggatgtatttggaattga
F431Q_reverse	tacatcctggtatgcagcatccc
N475C_forward	cgcttgtgtgtttacatagctgac
N475C_reverse	gtaaacacacaagcgagcgaaatc
S499A_forward (S-1’A)	ttggtggctgatattgggggtc
S499A_reverse (S-1’A)	caatatcagccaccaattgatttcttgtg
D500N_forward (D0’N)	gtgagtaatattgggggtcagc
D500N_reverse (D0’N)	ccaatattactcaccaattgatttcttgtg
G502S_forward (G2’S)	gtgatatttcgggtcagctaggtctc
G502S_reverse (G2’S)	ctgacccgaaatatcactcaccaattg
G503S_forward (G3’S)	attgggagtcagctaggtctctgg
G503S_reverse (G3’S)	ctagctgactcccaatatcactcacc
G506S_forward (G6’S)	cagctaagtctctgggtcggcatatc
G506S_reverse (G6’S)	ccagagacttagctgacccccaatatc
