# Supplementary material for: Structural basis for excitatory neuropeptide signaling
Source: Nat Struct Mol Biol. 2024 Feb 9;31(4):717–26. doi: 10.1038/s41594-023-01198-y (PMC11026163; doi:10.1038/s41594-023-01198-y)
Supplement: Supplementary file 2 — Reporting Summary [file 41594_2023_1198_MOESM2_ESM.pdf]

nature portfolio

Corresponding author(s): Timothy Lynagh, Cristina Paulino

Last updated by author(s): Nov 1, 2023

Reporting Summary

Nature Portfolio wishes to improve the reproducibility of the work that we publish. This form provides structure for consistency and transparency in reporting. For further information on Nature Portfolio policies, see our Editorial Policies and the Editorial Policy Checklist.

Statistics

For all statistical analyses, confirm that the following items are present in the figure legend, table legend, main text, or Methods section.

n/a	Confirmed	
☐	☒	The exact sample size (*n*) for each experimental group/condition, given as a discrete number and unit of measurement
☐	☒	A statement on whether measurements were taken from distinct samples or whether the same sample was measured repeatedly
☒	☐	The statistical test(s) used AND whether they are one- or two-sided<br>*Only common tests should be described solely by name; describe more complex techniques in the Methods section.*
☒	☐	A description of all covariates tested
☒	☐	A description of any assumptions or corrections, such as tests of normality and adjustment for multiple comparisons
☐	☒	A full description of the statistical parameters including central tendency (e.g. means) or other basic estimates (e.g. regression coefficient) AND variation (e.g. standard deviation) or associated estimates of uncertainty (e.g. confidence intervals)
☒	☐	For null hypothesis testing, the test statistic (e.g. *F*, *t*, *r*) with confidence intervals, effect sizes, degrees of freedom and *P* value noted<br>*Give P values as exact values whenever suitable.*
☒	☐	For Bayesian analysis, information on the choice of priors and Markov chain Monte Carlo settings
☒	☐	For hierarchical and complex designs, identification of the appropriate level for tests and full reporting of outcomes
☒	☐	Estimates of effect sizes (e.g. Cohen's *d*, Pearson's *r*), indicating how they were calculated

Our web collection on statistics for biologists contains articles on many of the points above.

Software and code

Policy information about availability of computer code

Data collection	SerialEM 3.9.0 beta for cryo-EM data collection. Patchmaster 2x90.4 (HEKA) for two electrode voltage clamp.
Data analysis	Cryo-EM (managed through SBGrid version 2.5.6): Focus 1.1.0, crYOLO 1.8.2, MotionCor2 1.4.0, CTFfind4.1.14, cryoSPARC v3, Relion 3.1.0, deepEMhancer 220530_cu10 for cryo-EM data processing. Alphafold 2, Coot 0.9.8.1, Chimera 1.16, Phenix 1.20.1-4487, Isolde 1.6.0 for cryo-EM model building and refinement. PyMol 2.5.5, ChimeraX v 1.5 for structure visualization.<br>Clampfit v11 for two electrode voltage clamp. Prism v9 for nonlinear regression analysis.

For manuscripts utilizing custom algorithms or software that are central to the research but not yet described in published literature, software must be made available to editors and reviewers. We strongly encourage code deposition in a community repository (e.g. GitHub). See the Nature Portfolio guidelines for submitting code & software for further information.

Data

Policy information about availability of data

All manuscripts must include a data availability statement. This statement should provide the following information, where applicable:

- Accession codes, unique identifiers, or web links for publicly available datasets
- A description of any restrictions on data availability
- For clinical datasets or third party data, please ensure that the statement adheres to our policy

Original Malacoceros fuliginosus FaNaC1 mRNA sequence available in Genbank ON156825.1 and utilized cDNA sequence available in reference 6 (https://

www.jbc.org/article/S0021-9258(22)00527-0/fulltext). Ligand-free, FMRFa-bound, ASSFVRIa-bound, and FMRFa-bound+diminazene structures available in PDB via entries 8ON8, 8ON7, 8ON9, and 8ONA, and in EMDB via entries 16982, 16981, 16983, and 16984 respectively. Micrographs were deposited to EMPIAR under the following accession codes: 11631 (apo FaNaC1), 11632 (FMRFa-bound FaNaC1), 11633 (ASSFVRIa-bound FaNaC1), and 11634 (FMRFa-bound FaNaC1 in the presence of diminazene). Source data for figures are available in the accompanying source data files.

Research involving human participants, their data, or biological material

Policy information about studies with human participants or human data. See also policy information about sex, gender (identity/presentation), and sexual orientation and race, ethnicity and racism.

Reporting on sex and gender	Not applicable.
Reporting on race, ethnicity, or other socially relevant groupings	Not applicable.
Population characteristics	Not applicable.
Recruitment	Not applicable.
Ethics oversight	Not applicable.

Note that full information on the approval of the study protocol must also be provided in the manuscript.

Field-specific reporting

Please select the one below that is the best fit for your research. If you are not sure, read the appropriate sections before making your selection.

☒ Life sciences ☐ Behavioural & social sciences ☐ Ecological, evolutionary & environmental sciences

For a reference copy of the document with all sections, see nature.com/documents/nr-reporting-summary-flat.pdf

Life sciences study design

All studies must disclose on these points even when the disclosure is negative.

Sample size	No statistical methods were used to predetermine sample size. Cryo-EM data collection was deemed sufficient if it was possible to achieve isotropic reconstructions at resolution 4Å or better for all of the datasets. For electrophysiology, experiments were generally performed on at least four different oocytes, as described in text. This is relatively standard in the field.
Data exclusions	Micrographs with the resolution 6Å or worse, displaying high drift, ice contamination or cracks, defocus values > 2 um and <0.3 um were excluded, according to the standard in the field. Further, particles not contributing to 2D classes with identifiable FaNaC1 features, and not contributing to the highest resolution map during 3D classification were excluded from subsequent processing. For electrophysiology, no exclusion criteria were established before experiments; obvious outliers (e.g. an oocyte showing no currents when all other oocytes in that condition showed currents) were excluded from analysis.
Replication	Protein was purified and reconstituted into lipid nanodiscs 3 times with similar results. Cryo-EM data collection and structure determination were performed once per dataset. The data was collected from 2 – 3 grids in all cases. For electrophysiology, all experiments were repeatable, e.g. across different oocytes, and also in different batches of oocytes.
Randomization	For cryo-EM, particles were randomized between even/odd groups during refinement and resolution estimation (gold-standard FSC). For electrophysiology, experiments were not performed in any particular order, so there was some "randomization", but there was no randomization in data analysis, as we were simply calculating means or pharmacological parameters.
Blinding	Blinding is not applicable in cryo-EM processing workflows as data are processed in an automated fashion, and for electrophysiology, we did not perform blinding, as we carefully labeled dishes of oocytes before recording from them.

Reporting for specific materials, systems and methods

We require information from authors about some types of materials, experimental systems and methods used in many studies. Here, indicate whether each material, system or method listed is relevant to your study. If you are not sure if a list item applies to your research, read the appropriate section before selecting a response.

Materials & experimental systems

n/a	Involved in the study
☐	☒ Antibodies
☐	☐ Eukaryotic cell lines
☐	☐ Palaeontology and archaeology
☐	☐ Animals and other organisms
☐	☐ Clinical data
☐	☐ Dual use research of concern
☐	☐ Plants

Methods

n/a	Involved in the study
☐	☐ ChIP-seq
☐	☐ Flow cytometry
☐	☐ MRI-based neuroimaging

Antibodies

Antibodies used	GFP enhancer nanobody, commonly called 3K1K
Validation	This is a sequenced nanobody, verified at length by Kirchhofer, A. et al. (2010) Nature Structural & Molecular Biology 17, 133-138.

Eukaryotic cell lines

Policy information about cell lines and Sex and Gender in Research

Cell line source(s)	HEK293T (CRL-3216) and HEK293S GnTI- (CRL-3022, ATCC) from American Tissue Culture Collection. Sf9 (12659017, ThermoFisher Scientific). Xenopus laevis frog unfertilized oocytes purchased from EcoCyte Bioscience, Germany.
Authentication	Cell lines were not authenticated after purchase.
Mycoplasma contamination	All cell lines were regularly tested for Mycoplasma contamination (every 3-4 months) and were found negative.
Commonly misidentified lines (See ICLAC register)	No commonly identified lines (according to ICLAC register) were used in this study.

Palaeontology and Archaeology

Specimen provenance	not applicable
Specimen deposition	not applicable
Dating methods	not applicable

☐ Tick this box to confirm that the raw and calibrated dates are available in the paper or in Supplementary Information.

Ethics oversight	not applicable

Note that full information on the approval of the study protocol must also be provided in the manuscript.

Animals and other research organisms

Policy information about studies involving animals; ARRIVE guidelines recommended for reporting animal research, and Sex and Gender in Research

Laboratory animals	No laboratory animals used.
Wild animals	No wild aninamals were used.
Reporting on sex	Not applicable
Field-collected samples	Not applicable
Ethics oversight	Not applicable

Note that full information on the approval of the study protocol must also be provided in the manuscript.

Clinical data

Policy information about clinical studies

All manuscripts should comply with the ICMJE guidelines for publication of clinical research and a completed CONSORT checklist must be included with all submissions.

Clinical trial registration	No clinical data used.
Study protocol	No clinical data used.
Data collection	No clinical data used.
Outcomes	No clinical data used.

Dual use research of concern

Policy information about dual use research of concern

Hazards

Could the accidental, deliberate or reckless misuse of agents or technologies generated in the work, or the application of information presented in the manuscript, pose a threat to:

No	Yes	
☒	☐	Public health
☒	☐	National security
☒	☐	Crops and/or livestock
☒	☐	Ecosystems
☒	☐	Any other significant area

Experiments of concern

Does the work involve any of these experiments of concern:

No	Yes	
☒	☐	Demonstrate how to render a vaccine ineffective
☒	☐	Confer resistance to therapeutically useful antibiotics or antiviral agents
☒	☐	Enhance the virulence of a pathogen or render a nonpathogen virulent
☒	☐	Increase transmissibility of a pathogen
☒	☐	Alter the host range of a pathogen
☒	☐	Enable evasion of diagnostic/detection modalities
☒	☐	Enable the weaponization of a biological agent or toxin
☒	☐	Any other potentially harmful combination of experiments and agents

Plants

Seed stocks	No plants used.
Novel plant genotypes	No plants used.
Authentication	No plants used.

ChIP-seq

Data deposition

☐ Confirm that both raw and final processed data have been deposited in a public database such as GEO.

☐ Confirm that you have deposited or provided access to graph files (e.g. BED files) for the called peaks.

Data access links<br>*May remain private before publication.*	*For "Initial submission" or "Revised version" documents, provide reviewer access links. For your "Final submission" document, provide a link to the deposited data.*
Files in database submission	*Provide a list of all files available in the database submission.*
Genome browser session<br>(e.g. UCSC)	*Provide a link to an anonymized genome browser session for "Initial submission" and "Revised version" documents only, to enable peer review. Write "no longer applicable" for "Final submission" documents.*

Methodology

Replicates	*Describe the experimental replicates, specifying number, type and replicate agreement.*
Sequencing depth	*Describe the sequencing depth for each experiment, providing the total number of reads, uniquely mapped reads, length of reads and whether they were paired- or single-end.*
Antibodies	*Describe the antibodies used for the ChIP-seq experiments; as applicable, provide supplier name, catalog number, clone name, and lot number.*
Peak calling parameters	*Specify the command line program and parameters used for read mapping and peak calling, including the ChIP, control and index files used.*
Data quality	*Describe the methods used to ensure data quality in full detail, including how many peaks are at FDR 5% and above 5-fold enrichment.*
Software	*Describe the software used to collect and analyze the ChIP-seq data. For custom code that has been deposited into a community repository, provide accession details.*

Flow Cytometry

Plots

Confirm that:

☐ The axis labels state the marker and fluorochrome used (e.g. CD4-FITC).

☐ The axis scales are clearly visible. Include numbers along axes only for bottom left plot of group (a 'group' is an analysis of identical markers).

☐ All plots are contour plots with outliers or pseudocolor plots.

☐ A numerical value for number of cells or percentage (with statistics) is provided.

Methodology

Sample preparation	No flow cytometry was used.
Instrument	No flow cytometry was used.
Software	No flow cytometry was used.
Cell population abundance	No flow cytometry was used.
Gating strategy	No flow cytometry was used.

☐ Tick this box to confirm that a figure exemplifying the gating strategy is provided in the Supplementary Information.

Magnetic resonance imaging

Experimental design

Design type	No MRI was used.
Design specifications	No MRI was used.

Behavioral performance measures	No MRI was used.

Acquisition

Imaging type(s)	No MRI was used.
Field strength	No MRI was used.
Sequence & imaging parameters	No MRI was used.
Area of acquisition	No MRI was used.

Diffusion MRI ☐ Used ☒ Not used

Preprocessing

Preprocessing software	No MRI was used.
Normalization	No MRI was used.
Normalization template	No MRI was used.
Noise and artifact removal	No MRI was used.
Volume censoring	No MRI was used.

Statistical modeling & inference

Model type and settings	No MRI was used.
Effect(s) tested	No MRI was used.

Specify type of analysis: ☐ Whole brain ☐ ROI-based ☐ Both

Statistic type for inference (See Eklund et al. 2016)	No MRI was used.
Correction	No MRI was used.

Models & analysis

n/a	Involved in the study
☒	☐ Functional and/or effective connectivity
☒	☐ Graph analysis
☒	☐ Multivariate modeling or predictive analysis
